# Characterization of Phosphate Solubilizing Bacterial Endophytes and Plant Growth Promotion In Vitro and in Greenhouse

**DOI:** 10.3390/microorganisms9091935

**Published:** 2021-09-11

**Authors:** Chuansheng Mei, Robert L. Chretien, B. Sajeewa Amaradasa, Yimeng He, Amy Turner, Scott Lowman

**Affiliations:** The Plant Endophyte Research Center, The Institute for Advanced Learning and Research, Danville, VA 24540, USA; Robert.Chretien@ialr.org (R.L.C.); Sajeewa.Amaradasa@ialr.org (B.S.A.); yimeng.he@ialr.org (Y.H.); amy.turner@ialr.org (A.T.); scott.lowman@ialr.org (S.L.)

**Keywords:** phosphate solubilizing bacteria, plant growth promotion, in vitro and greenhouse experiments, mechanisms for phosphate solubilization, gluconic acid production

## Abstract

Phosphate is one of the most important nutrients for plant growth and development, and only 0.1% of the phosphate in soils is available to plants. Currently, the use of excess phosphate fertilizer has caused surface and ground water pollution and water eutrophication, resulting in algal blooms in lakes and oceans. Therefore, it is imperative to explore alternative ways to solve these problems for sustainable agricultural production and improvement of soil fertility, while protecting the environment. Microorganisms from the rhizosphere and within plants are able to solubilize insoluble soil phosphate, making it available to plants. Five high phosphate solubilizing bacteria from our bacterial endophyte library were chosen for this study and identified as *Pantoea vagans* IALR611, *Pseudomonas psychrotolerans* IALR632, *Bacillus subtilis* IALR1033, *Bacillus* *safensis* IALR1035 and *Pantoea agglomerans* IALR1325. All five bacteria significantly promoted tall fescue growth in vitro. Greenhouse experiments showed that IALR1325 significantly promoted pepper and tomato growth, and IALR632 was the best in promoting tomato growth. In addition, all these bacteria had extracellular acid phosphatase and phytase activities. One of the mechanisms for phosphate solubilization by bacteria is pH reduction caused by gluconic acid production. Our results indicate that *P. agglomerans* IALR1325 is a promising bacterium for future applications.

## 1. Introduction

Phosphate is one of the most important nutrients for plant growth and development and plays important roles in all metabolic processes [1,2]. Phosphate is abundant in soil but is mostly insoluble due to P fixation [3], which causes P deficiency. Only 0.1% of phosphate in soils is available to plants [4]. Currently, in order to increase yields, the use of excess phosphate fertilizer has caused surface and ground water pollution and promoted water eutrophication due to running off, which results in algal blooms and adversely affects water quality. 

For instance, the devastating algal bloom in the Lake Erie in Toledo, OH, USA in 2014 cut off drinking water for 500,000 people [5]. Moreover, most soluble chemical P fertilizers can be fixed with Ca^2+^, Fe^3+^ and Al^3+^ to form insoluble calcium phosphate, ferric phosphate and aluminum phosphate, respectively, which leads to the rapid formation of poorly available P for plants after fertilizer application [6]. Therefore, there is a dilemma with respect to phosphate in soil. The total phosphate content is abundant in soil; however, only a minor amount is available to plants.

There have been many reports that microorganisms in the rhizosphere and within plants (endophytes) are able to solubilize insoluble soil phosphate, making it available to plants [1,2,7]. For example, Song et al. (2008) isolated a mineral phosphate solubilizing bacterium, *Burkholderia cepacia* DA23 from agricultural soil and demonstrated that DA23 had a higher ability to solubilize calcium phosphate and hydroxyapatite, but a minimal ability for aluminum phosphate [8]. Poonguzhali et al. (2008) isolated phosphate solubilizing bacteria from rhizosphere, which promoted the growth of Chinese cabbage [9]. Sharon et al. (2016) reported that *Pantoea* sp. Pot1 could solubilize calcium phosphate at a high rate and enhance tomato plant growth in greenhouse conditions [10]. Recently, it was reported that phosphate solubilizing bacterial endophytes *Enterobacter* sp. J49 or *Serratia* sp. S119 from peanut plants significantly promoted soybean and maize plant growth on a microcosm scale [11], which indicated that phosphate solubilizing bacteria could be used in different plant species for improving phosphate use efficiency.

The interest to reduce chemical P fertilizer in sustainable agricultural production is to find an alternative and inexpensive technology that could provide sufficient P to plants while reducing the dependence on expensive chemical P fertilizers [7]. Plant-associated, phosphate solubilizing bacteria are one of the alternative ways to replace chemical P fertilizer. Therefore, it is imperative to explore phosphate solubilizing microorganisms for sustainable agricultural production and the improvement of soil fertility to protect the environment from excess P. In past decades, there were increasing investigations in this area but with a little application, partly due to lack of knowledge about phosphate solubilizing bacteria.

Acid phosphatases catalyze the hydrolysis of phosphomonoesters at an acidic pH [12] to release P from organic matter. Phytases specifically release P from phytate, which is a major form of stored organic phosphorus compounds [13]. The process is predominantly catalyzed by phytase enzymes secreted by microorganisms and makes P available to plants. These enzymes play important roles in soil organic phosphate recycling, and the microorganisms that produce these enzymes assist plants by acquiring P from organic matter in soil [14].

The mechanisms for bacteria solubilizing insoluble phosphate are not completely understood. pH reduction caused by organic acid production is one of main mechanisms for bacteria to solubilize insoluble phosphate [1,7]. The pH reduction acidifies the surroundings, and H^+^ replaces Ca^2+^ to release the phosphate ion [1]. Gluconic acid is one of the organic acids produced by the phosphate solubilizing bacteria and chelates the cations bound to phosphate. There have been reports that some Gram-negative bacteria could oxidize glucose to gluconic acid directly [15].

The first and key enzyme in the direct oxidation of glucose to gluconic acid is glucose dehydrogenase. Other mechanisms for mineral phosphate solubilization by bacteria are the production of inorganic acids and the production of chelating substances [1]. In this study, we first screened phosphate solubilizing bacteria from our bacterial endophyte library, then characterized other plant growth promoting traits, tested plant growth promotion with insoluble phosphate in vitro and in greenhouse, and finally studied their mechanisms with organic acid measurements and enzymatic assays.

## 2. Materials and Methods

### 2.1. Isolation of Bacterial Endophytes

Bacterial endophytes were isolated from plants grown in the foothills of the Appalachian Mountains in Central Virginia, USA (geographic location: 37.125372, −79.298415). The soil was not fertilized. Healthy plants were selected randomly from the natural environment. Plants were taken from the field and brought to the lab for isolation or kept in a refrigerator temporarily. The plants were first washed with tap water to remove the soil, and then surface was sterilized with 5% Clorox^®^ bleach (The Clorox Company, Oakland, CA, USA) containing 8.5% NaOCl with a drop of Tween 20 for 5 min. Finally, the plants were rinsed with sterile water five times. 

To confirm the sterilization efficacy, 50 µL of the last rinsate were plated on Luria agar (LA) medium. The surface-sterilized plant was divided into the root, leaf and stem, which were ground individually with sterile water. The ground tissues were centrifuged at 2000 rpm for 3 min to remove the debris. The supernatant was diluted to 10 ×, 100 × and 1000 × with sterile water, and then 50 µL were spread on LA plates. The plates were placed at 28 °C for 2–5 days. Different individual colonies were streaked onto a fresh LA plate for purification and for producing a glycerol stock for long-term storage.

### 2.2. Screening Phosphate Solubilizing Bacteria by Quantifying Soluble P in Medium

Isolated bacteria were screened for phosphate solubilizing ability as follows. Overnight bacterial culture (0.1 mL) at ~1.5 of OD_600_ was added to 3.9 mL of NBRIP [16] with 0.5% of calcium phosphate [Ca_3_(PO_4_)_2_], hydroxyapatite [Ca_5_(PO_4_)_3_OH], aluminum phosphate (AlPO_4_) or ferric phosphate (FePO_4_) (Sigma-Aldrich, St. Louis, MO, USA), and incubated at 28 °C with shaking at 200 rpm for 3 days. One mL of bacterial cultures was centrifuged at 14,000 rpm for 10 min, and the soluble P in the supernatant was determined with the method of Murphy and Riley [17]. The amount of soluble P was calculated from the standard curve of P concentration using KH_2_PO_4_.

### 2.3. Acid Phosphatase and Phytase Activity Assays

Overnight bacterial culture (0.4 mL) was added to a 125 mL of flask containing 20 mL of NBRIP with 0.5% of calcium phosphate and incubated at 28 °C with shaking at 200 rpm for 4 days in triplicate for each bacterium. Bacterial cultures were centrifuged at 5000 rpm at 4 °C for 5 min, and supernatant was added with 3 volumes of cold ethanol. The protein fraction was precipitated by centrifugation (5000 rpm, 20 min, 4 °C), and 100 mM sodium acetate buffer (pH 6.0) was added to suspend the pellet, which was considered to be extracellular enzymes from bacteria. 

Acid phosphatase assay followed the protocol of Rombola et al. [18] to quantify the amount of 4-nitrophenol released by acid phosphatase with measurement at 405 nm. The protein concentration was determined with Bio-Rad Protein Dye Solution (Bio-Rad, Hercules, CA, USA) with BSA as standard. The specific enzyme activity was defined as µg 4-nitrophenol released by enzyme per mg protein per hour. A phytase activity assay followed the protocol of Parhamfar et al. [19] to measure the P amount released from phytic acid by phytase activity with a color reagent (1 part of 2.7% FeSO_4_ and 4 parts of 1.5% ammonium molybdate in 4.4% H_2_SO_4_) at OD_700_ using K_2_HPO_4_ as the P standard. The specific enzyme activity was the µg P released by the enzyme per mg protein per hour.

### 2.4. Other Plant Growth Promoting Traits of Phosphate Solubilizing Bacteria

#### 2.4.1. Auxin Quantification

The auxin quantification method was modified from Patten and Glick’s protocol [20] following our previous publication [21] using Salkowski reagent with bacterial growth in LB containing 1 g/L tryptophan for 3 days. Indole acetic acid (IAA) was used for a standard curve. The auxin concentration was expressed as μg/mL of bacterial culture.

#### 2.4.2. ACC Deaminase Activity Screening

Aminocyclopropane-1-carboxylate (ACC) deaminase activity screening was conducted with bacterial cultures grown in LB medium overnight. Ten µL were then added to 2 mL DF medium containing 3 mM ACC as the sole nitrogen source. *Burkholderia phytofirmans* strain PsJN was used as a positive control because it has a high ACC deaminase activity [22]. Bacterial endophyte cultures were incubated for 6 days at 28 °C with 200 rpm. The absorbance at 600 nm was recorded for bacterial growth. The ranking was as follows: equal to that of PsJN, ++; greater than that of PsJN, +++; less than that of PsJN, +; and no bacterial growth was ranked as −.

#### 2.4.3. Siderophore Assay

The siderophore assay was modified based on the protocol of Payne [23]. Bacterial endophytes were cultured in LB medium overnight. Ten µL were then added to 2 mL of King’s B medium without P and incubated for 5 days at 28 °C with shaking at 200 rpm. One mL of bacterial culture was centrifuged at 14,000 rpm for 5 min, and 0.5 mL of supernatant was mixed with 0.5 mL of CAS assay solution and incubated at room temperature for 20 min. The medium without bacterial inoculation was used as a reference. Then, the absorbance was measured at 630 nm. Siderophore units were defined as: % siderophore units = [(Ar − As)/Ar] × 100%. The sample absorbance, denoted as “As”, is supposed to be lower than that of the reference, denoted as “Ar”.

#### 2.4.4. N Fixation Screening

N fixation screening followed the protocol of Pathak and Kalekar [24]. Briefly, overnight bacterial cultures were streaked in Norris N free medium with glucose as the carbon source and incubated at 28 °C in an incubator for 5 days. The bacterial growth was recorded. Those that grew were streaked in fresh Norris N free medium for another two times, and growth was marked as + and no growth as −.

### 2.5. Identification of Phosphate Solubilizing Bacteria and Phylogenetic Tree Construction

Based on the phosphate solubilizing ability, five bacteria (strains IALR611, IALR632, IALR1033, IALR1035 and IALR1325) were selected for identification with 16S rRNA gene sequencing by Genewiz Company (South Plainfield, NJ, USA). The 16S rRNA sequences of the five strains were used to construct a phylogenetic tree with GenBank deposited reference sequences. Multiple sequence alignment and tree building was performed in MEGA software [25].

### 2.6. pH Measurement and Gluconic Acid Quantification

We inoculated 0.5 mL of bacterial overnight cultures at ~1.5 of OD_600_ into 25 mL of NBRIP with 0.5% of calcium phosphate in 125 mL of flask and incubated at 28 °C with shaking at 200 rpm for 4 days. The pH values were recorded with a pH meter. The bacterial cultures were centrifuged at 14,000 rpm for 10 min. The supernatant was filtered with a 0.2 µm filter column for HPLC injection. Gluconic acid was quantified using an Agilent 1100 HPLC (Agilent Technologies, Santa Clara, CA, USA) equipped with a quaternary pump, autosampler, DAD detector and degasser. 

The chromatographic separation of gluconic acid was achieved at ambient temperature using a 300 × 4.6 mm ID, 5 µm particle size Allure Organic Acids column (Restek, Bellefonte, PA, USA). Isocratic elution was carried out using a mobile phase of 100 mM phosphate buffer at 0.5 mL/min, and the retention time was 6.4 min with a 10 μL injection. A UV/Vis detector at 210 nm wavelength was used for detection. Pure gluconic acid was purchased from Sigma-Aldrich (St. Louis, MO, USA) and used as a reference.

### 2.7. Plant Growth Promotion In Vitro

The protocol for growth promotion in vitro followed our previous publication [21]. Briefly, tall fescue (*Festuca arundinacea*) KY 31 seeds without fungal endophytes (kindly provided by Dr. Chris Teutsch) were rinsed with 70% ethanol for 2 min, and their husks were removed with 60% H_2_SO_4_ for 35 min, rinsed with tap water, then surface-sterilized with 50% Clorox^®^ bleach for 35 min and finally rinsed with sterile water five times. The seeds were germinated on wet filter paper in petri-dishes for 4–6 days at 25 °C under white fluorescent light (50 μmol m^−2^ s^−1^) with a 16 h photoperiod. 

The root tips of the young seedlings were cut prior to bacterial inoculation to facilitate bacterial penetration, and the seedlings were inoculated by soaking in bacterial solution (OD_600_ = 0.5) for 1 min. The control seedlings were treated with PBS buffer alone. A total of 25 seedlings were used for each bacterial treatment. The treated seedlings were blot-dried with sterile paper towel, placed in GA7 Magenta vessels (Sigma-Aldrich, St. Louis, MO, USA) containing 50 mL of medium (0.61 g/L M407 from PhytoTechnology Laboratory, Lenexa KS, 1 g/L Ca_3_(PO_4_)_2_, 1.65 g/L (NH_4_)_2_SO_4_, 1.9 g/L KNO_3_, 10 g/L maltose, 0.3% gelrite, pH 5.8). In each vessel, five seedlings were placed and grown under the same conditions as above. After 6 weeks, the seedling fresh weight was determined.

### 2.8. Plant Growth Promotion in Greenhouse

As we are more interested in applying phosphate solubilizing bacteria in vegetable crops, tomato (*Solanum lycopersicum*) cv. Beefsteak and pepper (*Capsicum annuum*) cv. California Wonder plants were tested for growth promotion by the above strains in a greenhouse with randomized complete block design with eight blocks and three plants per block. Seeds were germinated in 1.5 Rockwool Starter Plugs in an incubator (as described above). The seedlings were transferred to sand/clay pebble (1:1) in greenhouse, and 1 mL of freshly growing bacteria (OD_600_ = 1.0) per seedling was inoculated around the root area the next day. The seedlings were then watered with ¼ strength of the nutrient solution (0.61 g/L M407, 0.5 g/L Ca_3_(PO_4_)_2_, 0.825 g/L (NH_4_)_2_SO_4_, 0.95 g/L KNO_3_, pH 5.8). The dry weights of the roots and shoots were determined after one month of growth.

### 2.9. Statistical Analyses

For growth promotion in vitro, a completely randomized design was used. For growth promotion in greenhouse, a randomized complete block design was applied. The SAS Pearson correlation coefficient test was conducted for the gluconic acid contents and pH values. Statistical analyses were conducted with one-way ANOVA, and least significant difference (LSD) was used for multiple treatment comparison using SAS^®^ Studio from SAS OnDemand for Academics. All significant levels were set at *p* < 0.05.

## 3. Results

### 3.1. Identification of Phosphate Solubilizing Bacteria and Phylogenetic Tree

**Figure 1:**
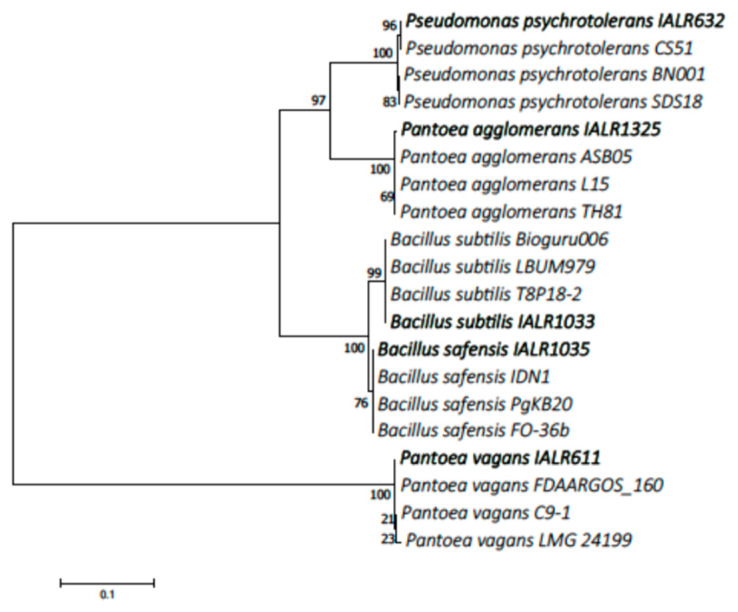
Phylogenetic tree for the phosphate solubilizing bacteria with GenBank references. The tree was constructed using neighbor-joining method with bootstrap support values derived from 1000 replications and shown as a percentage next to the branches. The bacterial strains used in this study were highlighted in bold.

Based on our preliminary results for phosphate solubilization and 16S rRNA sequences, five high phosphate solubilizing bacteria representing different species were chosen for further study. The information of origination and species identification are listed in Table 1. These bacterial 16S rRNA sequences were submitted to GenBank, and the accession numbers are listed in Table 1. The phylogenetic tree constructed with verified bacterial species from GenBank confirmed the species level identity of bacterial endophytes used in this study (Figure 1). A BLAST search of the reference GenBank isolates in the tree matched > 99% to our 16S nucleotide sequences.

### 3.2. Phosphate Solubilizing Ability of Bacteria

Different insoluble phosphate compounds were tested for solubilization by these bacteria over 3 days, and the results are listed in Table 2. Overall, these bacteria had the highest solubilizing ability for calcium phosphate (Ca_3_(PO_4_)_2_) and a relatively high ability for hydroxyapatite [Ca_5_(PO_4_)_3_OH], but a minimal ability for AlPO_4_ and FePO_4_. These results may be due to the degrees of solubility of these phosphate compounds. Calcium phosphate has a high degree of solubilization in water (1.2 mg/L) while AlPO_4_ and FePO_4_ are insoluble in water.

### 3.3. Activities of Acid Phosphatase and Phytase of Phosphate Solubilizing Bacteria

The results of the acid phosphatase and phytase activities indicated that these bacteria secrete these enzymes outside the cells (Table 3). *P. psychrotolerans* IALR632 had the highest acid phosphatase activity, 95–375% higher than other bacteria did, while it had the lowest phytase activity.

### 3.4. Other Plant Growth Promoting Traits of the Phosphate Solubilizing Bacteria

In addition to phosphate solubilization, the bacterial strains had other growth promoting traits (Table 4). All of the strains had siderophore activity. Two of the strains (*P. vagans* IALR611 and *P. agglomerans* IALR1325) produced high levels of auxins. Three had ACC deaminase activity, and three had possible nitrogen fixing ability (Table 4).

### 3.5. pH Changes and Gluconic Acid Production

**Figure 2:**
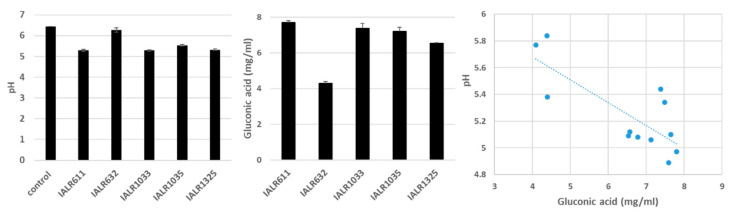
pH changes and gluconic acid production by bacteria after 4 days of culture.

The pH values of the medium were reduced from 6.0 in the control to 4.9 in IALR611, after 4 days. Gluconic acid concentrations were abundant in the medium after 4 days of bacterial growth (Figure 2). With the SAS Pearson correlation test, we found that there was a negative relationship between the gluconic acid concentrations and pH values (correlation coefficient *R* = −0.78218; *p* = 0.0026).

### 3.6. Plant Growth Promotion In Vitro

**Figure 3:**
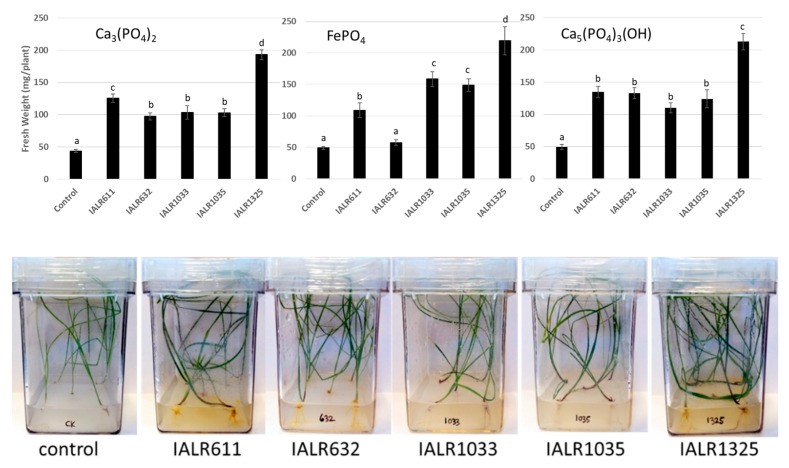
Top: tall fescue plant growth promotion by phosphate solubilizing bacteria in vitro with different insoluble phosphate compounds. Bottom: comparison of bacterium inoculated plants with control plants using Ca_3_(PO_4_)_2_ as the P source in the medium. Different letters on bars mean significantly different at *p* < 0.05.

In in vitro experiments with Ca_3_(PO_4_)_2_ for about 6 weeks, all five bacteria significantly promoted growth, compared to the non-inoculated controls. The tall fescue KY31 growth increased by 185%, 121%, 135%, 134% and 339% for IALR611, IALR632, IALR1033, IALR1035 and IALR1325, respectively. Moreover, these bacteria also significantly promoted tall fescue plant growth with ferric phosphate and hydroxyapatite as a P source, except IALR632 with FePO_4_ (Figure 3). Among these bacteria, IALR1325 showed the best growth promotion in vitro.

### 3.7. Growth Promotion by Phosphate Solubilizing Bacteria in Greenhouse Experiments

**Figure 4:**
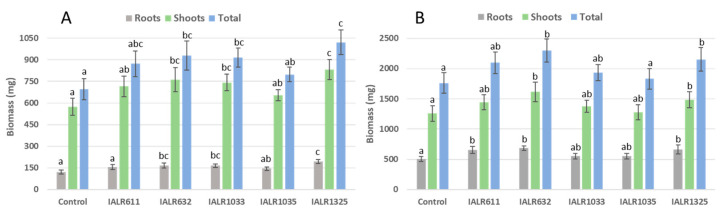
Phosphate solubilizing bacteria promoted the growth of pepper (**A**) and tomato (**B**) plants. Different letters on bars mean significantly different at *p* < 0.05.

These phosphate solubilizing bacteria were then tested for growth promotion of tomato and pepper plants in greenhouse conditions (Figure 4). The inoculation of IALR1325, IALR632 and IALR1033 significantly promoted the plant growth of peppers, with a 46.8%, 33.6% and 31.4% increase in total biomass, respectively, compared with the control treatment. The inoculation of IALR632 and IALR1325 significantly increased the biomass of the shoots and roots of tomato plants, with a 30.5% and 22.2% increase, respectively, compared with the control treatment. Other treatments showed plant growth promotion; however, the differences were not statistically significant from the control.

## 4. Discussion

Phosphate is one of the most important nutrients for plant growth and development, next to nitrogen. The total phosphate content in soil is abundant, while the phosphate available to plants is very low because phosphate is chelated with various cations, such as Ca^2+^, Al^3+^ and Fe^3+^. Once phosphate fertilizer is applied, it quickly becomes unavailable to plants or runs off, posing a risk of water contamination [26]. Another P source in soil is organic phosphates from plant residues. 

Phytate is a major storage form of organic phosphate, which is also unavailable for plant use because it also forms complexes with cations [27]. Many bacteria from the rhizosphere have phytase activity, which could release soluble phosphate from phytate [28]. In order to solve low phosphate availability, excess phosphate fertilizer has been used, which causes environmental contamination as well as increases farmer input. Phosphate solubilizing bacteria can release P available to plants [3]. In this study, we chose five different bacterial isolates from our bacterial endophyte library, which are able to solubilize insoluble phosphate compounds. 

Our results showed that these bacteria could efficiently solubilize calcium phosphate and hydroxyapatite but had only a minimal ability to solubilize ferric phosphate and aluminum phosphate during bacterial growth in LB medium (Table 2). Interestingly, these endophytes were isolated from shoots, leaves or seeds. We surveyed our bacterial endophyte library and found that 58% of phosphate solubilizing bacteria came from aboveground tissues and 42% from roots (data not shown). Oteino et al. reported that the two highest phosphate solubilizing bacteria were isolated from leaf tissues [29]. Recently, Chen et al. described that one bacteria from stem tissue had high phosphate solubilizing ability and increased the growth of Chinese fir seedlings [30]. Bacterial endophytes play important roles in phosphate solubilization for plant growth and development directly or indirectly and incorporate the rhizobia [31]. More importantly, Varga et al. suggested that endophytes might help in the re-release of phosphate because the solubilized phosphate may become insoluble inside plant tissue [32].

With plant growth promotion in vitro, these phosphate solubilizing bacteria significantly promoted tall fescue plant growth with calcium phosphate and hydroxyapatite as the sole phosphate source (Figure 2). Although these bacteria had a minimal ability to solubilize FePO_4_ in bacterial cultures, the inoculation of these bacteria greatly increased the tall fescue plant growth in in vitro experiments with FePO_4_ as the sole P source in medium. The results may also be attributed to other plant growth promoting traits, such as auxins and siderophore production and nitrogen fixation by these bacteria (Table 2). 

In these experiments, P may not be the limiting factor during plant growth, which might be resulted in by plant and bacterium interactions. More work needs to be done in determining the P available in the medium during plant growth with or without bacterial inoculation and how much P is required for the maximum plant growth. In the in vitro experiments, there was a greater than 300% increase in fresh weight by IALR1325. 

The greenhouse experiments also showed that IALR1325 significantly promoted both pepper and tomato plant growth, but to a lesser degree (Figure 4), which agrees with many other reports due to complex environmental conditions interfering with the phosphate solubilizing activity. From both in vitro and greenhouse experiments, *P. agglomerans* IALR1325 was the best bacterium for future potential applications due to the phosphate solubilization ability and other plant growth promoting traits, such as auxin and siderophore production as well as N fixation.

Mechanisms for phosphate solubilizing bacteria to solubilize phosphate compounds have been reported including organic acid production by bacteria to lower pH [3]. Gluconic acid is one of the most prevalent organic acids produced by phosphate solubilizing bacteria and fungi [33,34,35]. We quantified gluconic acid in bacterial culture medium using HPLC and detected large amount of gluconic acid due to bacterial growth (Figure 2), which agreed with the results of Vyas and Gulati [34] and Oteino et al. [29]. 

We found that there was a negative relationship between gluconic acid contents and pH values, which further confirms pH reduction caused by organic acid production by phosphate solubilizing bacteria. Oteino et al. obtained a similar result that the more gluconic acid production, the higher P released by their bacteria and suggested that acidification seemed to be a major mechanism for the phosphate solubilizing bacteria [29]. The reason for the large amount of gluconic acid production is that the medium (NBRIP) has 1% glucose, which is directly oxidized to gluconic acid by glucose dehydrogenases produced by bacteria. 

We found that all five bacteria had glucose dehydrogenase genes, which encode an enzyme in the direct oxidation pathway of glucose to produce gluconic acid from whole genome sequence analysis (data not shown). In addition, their genomes encode for the biosynthetic machinery for pyrroloquinoline quinone (PQQ) production. The cofactor PQQ is required by the glucose dehydrogenase enzyme for its function [12]. Oteino et al. also found that three leaf endophytic bacteria possessed a full pqq operon and glucose dehydrogenases by genetic analysis [29].

Acid phosphatase, secreted by phosphate solubilizing bacteria, can mineralize phosphate from organic compounds. Phytase specifically releases phosphate from phytic acid, a major storage form of organic phosphate. Our results indicated that phosphate solubilizing bacteria have both acid phosphatase and phytase activities in agreement with medium acidification by gluconic acid, which would benefit plants to obtain available P from soil and plant residues. However, these bacteria do not have extracellular alkaline phosphatase activity (data not shown).

Phosphate solubilizing bacteria can effectively solubilize and mineralize inorganic and organic phosphate compounds and make them available to plants from soils and residues. However, the successful application of phosphate solubilizing bacteria in sustainable agricultural production is still in the early stages of development. Further research needs to be done for potential phosphate solubilizing bacteria in field trials, including different soil types, crops, the viability of phosphate solubilizing bacteria in soils, methods for application, competition with other microorganisms in soils, and the health of the soil microbial community.

## Figures and Tables

**Table 1:** Origination and identification of the phosphate solubilizing bacteria.

Strains	Species	Accession Number	Tissues	Plants
IALR611	*Pantoea vagans*	MZ519966	Shoot	*Taraxacum officinale*
IALR632	*Pseudomonas psychrotolerans*	MZ519967	Leaf	*Sorghum halepense*
IALR1033	*Bacillus subtilis*	MZ519968	Leaf	*Ambrosia trifida*
IALR1035	*Bacillus sufensis*	MZ519969	Leaf	*Viola sororia*
IALR1325	*Pantoea agglomerans*	MZ519970	Seed	*Festuca arundinacea*

**Table 2:** The solubilization of different insoluble phosphate compounds by bacterial endophytes.

Phosphate Compounds	*Pantoea vagans* IALR611	*Pseudomonas psychrotolerans* IALR632	*Bacillus* *subtilis* IALR1033	*Bacillus* *safensis* IALR1035	*Pantoea* *agglomerans* IALR1325
Ca_3_(PO_4_)_2_	284.5 ± 10.7 ^1^	289.3 ± 23.7	349.7 ± 23.1	274.6 ± 15.2	372.8 ± 11.9
Ca_5_(PO_4_)_3_OH	118.2 ± 21.7	126.0 ± 5.5	105.2 ± 16.9	118.0 ± 14.4	146.2 ± 13.1
AlPO_4_	4.6 ± 0.2	1.3 ± 0.6	6.0 ± 0.8	4.3 ± 1.3	9.1 ± 0.3
FePO_4_	7.8 ± 0.1	6.5 ± 0.4	8.3 ± 0.1	8.1 ± 0.1	9.2 ± 0.4

^1^ P content (µg/mL) in bacterial cultures after 3-day incubation and data from the average of three replicates ± standard errors. All data were calculated by subtracting baseline P release in sterile medium.

**Table 3:** Acid phosphatase and phytase activities produced by bacterial endophytes.

Enzyme Specific Activities	*Pantoea* *vagans* IALR611	*Pseudomonas* *psychrotolerans* IALR632	*Bacillus* *subtilis* IALR1033	*Bacillus* *safensis* IALR1035	*Pantoea* *agglomerans* IALR1325
Acidphosphatase	152.0 ± 7.1	721.9 ± 8.8	195.7 ± 3.9	198.8 ± 14.4	369.4 ± 16.3
Phytase	139.6 ± 21.9	64.7 ± 18.2	113.1 ± 27.4	211.3 ± 45.7	138.2 ± 29.1

**Table 4:** Other plant growth promoting traits of phosphate solubilizing bacteria.

Plant Growth Promoting Traits	*Pantoea* *vagans* IALR611	*Pseudomonas* *psychrotolerans* IALR632	*Bacillus* *subtilis* IALR1033	*Bacillus* *safensis* IALR1035	*Pantoea* *agglomerans* IALR1325
Auxins (µg/mL)	106.3 ± 10.5	13.4 ± 1.8	1.9 ± 0.6	0	87.3 ± 6.3
N Fixation	+	+	−	−	+
ACC deaminase	++	++	+	−	−
Siderophore (%)	89 ± 1.6	97 ± 0.5	58 ± 2.2	50 ± 9.9	66 ± 5.5

Note: All data were calculated by subtracting baseline data in sterile medium.
